# Non-invasive detection of adeno-associated viral gene transfer using a genetically encoded CEST-MRI reporter gene in the murine heart

**DOI:** 10.1038/s41598-018-22993-4

**Published:** 2018-03-15

**Authors:** Shelby Meier, Assaf A. Gilad, J. Anthony Brandon, Chenghao Qian, Erhe Gao, Jose F. Abisambra, Moriel Vandsburger

**Affiliations:** 1 Department of Physiology, University of Kentucky, Lexington, KY USA; 2 Sanders Brown Center on Aging, University of Kentucky, Lexington, KY USA; 3 Department of Radiology, Johns Hopkins University, Baltimore, MD USA; 4 The Institute for Cell Engineering, Johns Hopkins University, Baltimore, MD USA; 5 Department of Biomedical Engineering, Michigan State University, East Lansing, MI USA; 6 The Institute of Quantitative Health Science and Engineering, Michigan State University, East Lansing, MI USA; 7 Department of Radiology, Michigan State University, East Lansing, MI USA; 8 Center for Translational Medicine, Temple University, Philadelphia, PA USA; 9 Department of Bioengineering, University of California, Berkeley, CA USA

## Abstract

Research into gene therapy for heart failure has gained renewed interest as a result of improved safety and availability of adeno-associated viral vectors (AAV). While magnetic resonance imaging (MRI) is standard for functional assessment of gene therapy outcomes, quantitation of gene transfer/expression relies upon tissue biopsy, fluorescence or nuclear imaging. Imaging of gene expression through the use of genetically encoded chemical exchange saturation transfer (CEST)-MRI reporter genes could be combined with clinical cardiac MRI methods to comprehensively probe therapeutic gene expression and subsequent outcomes. The CEST-MRI reporter gene Lysine Rich Protein (LRP) was cloned into an AAV9 vector and either administered systemically via tail vein injection or directly injected into the left ventricular free wall of mice. Longitudinal *in vivo* CEST-MRI performed at days 15 and 45 after direct injection or at 1, 60 and 90 days after systemic injection revealed robust CEST contrast in myocardium that was later confirmed to express LRP by immunostaining. Ventricular structure and function were not impacted by expression of LRP in either study arm. The ability to quantify and link therapeutic gene expression to functional outcomes can provide rich data for further development of gene therapy for heart failure.

## Introduction

Improved safety profiles and the availability of multiple serotypes of adeno-associated viral vectors (AAV) with distinct organ affinity^1^ have renewed interest in gene therapy approaches, particularly for cardiovascular disease^2,3^. Early clinical trials including the calcium upregulation by percutaneous administration of gene therapy in patients with cardiac disease (CUPID) trial that sought to restore cardiac function in heart failure patients via AAV1 mediated gene delivery often showed promise in early stage trials^4^ but did not provide definitive benefit in larger phase II trials^5–7^. Immuno-histochemical evidence from individuals who proceeded to cardiac transplantation confirmed inadequate therapeutic gene expression in non-responders^6,7^. Important methodological questions including whether a different delivery scheme (e.g. route, dose, repeated administration, concentration of empty capsids) would have resulted in increased therapeutic gene expression, in terms of both magnitude and spatial distribution, and subsequent outcomes in such individuals remain unanswered. The ability to repeatedly quantify and correlate the degree and distribution of therapeutic gene expression to functional outcomes on an individual basis would provide a rich data set that can be used to refine the preparation, execution, and continued monitoring in cardiac gene therapy.

Quantitative measurement of gene expression following viral delivery is has been performed using fluorescent/ bioluminescent whole body imaging in small animals^1,8,9^. However, such techniques are not scalable to large animal models or clinical settings. Alternative reporter imaging techniques including sodium-iodide (Na/I) transporter imaging using single photon emission computed tomography (SPECT) require radioactive probes^10^. Subsequently, clinical monitoring of gene expression patterns requires either undesirable repeated radiation exposure or extrapolation from repeated biopsies^5^. Both requirements represent a substantial barrier in individuals with heart failure that can be addressed by non-invasive imaging. Magnetic resonance imaging (MRI) is used clinically for assessment and quantification of cardiac structure and function^11^. Additionally, quantitative MRI techniques are now routinely used to measure important outcomes of gene therapy including tissue perfusion, contractile function, and fibrosis^12^. Molecular MRI with chemical exchange saturation transfer (CEST) is an emerging approach for the multi-color detection of molecular probes and reporter genes that are selectively imaged with tunable radiofrequency energy at distinct resonant frequencies^13–15^. Recently a number of CEST active genetically encoded reporter proteins^16–20^ including the Lysine Rich Protein (LRP), which is comprised of 200 lysine residues and generates CEST contrast when excited with RF energy tuned to 3.76 parts per million (ppm) offset from water, have been developed and utilized for *in vivo* cell tracking and for quantitative imaging of oncolytic virotherapy in stationary organs^18,21,22^.

The application of genetically encoded CEST-MRI reporters for imaging in cardiac gene therapy could enable the immediate superimposition of quantitative *in vivo* imaging of gene expression patterns with functional outcomes at the spatial resolution of MRI and without the need for biopsy or radioactive tracers. We have developed and applied a specialized cardiac CEST-MRI approach for non-invasive imaging of fibrosis^23^, creatine metabolism^24^, and for quantitative cardiac cell tracking in small animals^24,25^. In the current study we cloned the Lysine Rich Protein into an AAV9 vector and measured regional and time dependent changes in CEST-MRI contrast following either direct injection into the ventricular wall, or systemic administration via the tail vein.

## Results

### Cell Transfection

**Figure 1:**
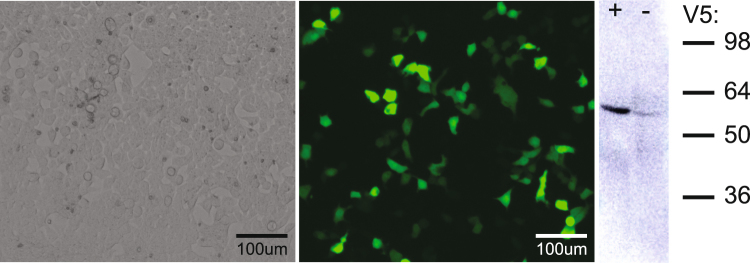
Transfection of HEK293T cells using the AAV9 vector encoding for eGFP, V5 and LRP. (Left) Phase contrast image of HEK293T cells and (middle) corresponding fluorescence microscopy image showing expression of eGFP. (Right) Western blot of cell homogenates for cells infected with AAV9-LRP (+) or control uninfected cells (−) demonstrates positive staining for the V5 epitope. The position of the LRP-V5 band with respect to the ladder is influenced by the strongly positive charge of LRP compared to other proteins of similar size.

The reporter gene construct contained the Lysine Rich Protein, enhanced green fluorescent protein (eGFP), and the V5 tag under control of the CMV promoter (pAAV9-CMV_prom_::LRP-IRES_eGFP-V5). Transfection of isolated HEK293T cells resulted in green fluorescence of transfected cells (Fig. 1). Western blot analysis of homogenized cells revealed strong staining for V5 in cells transfected with the AAV9 construct.

### Direct injection of AAV9-LRP regionally elevates CEST contrast

**Figure 2:**
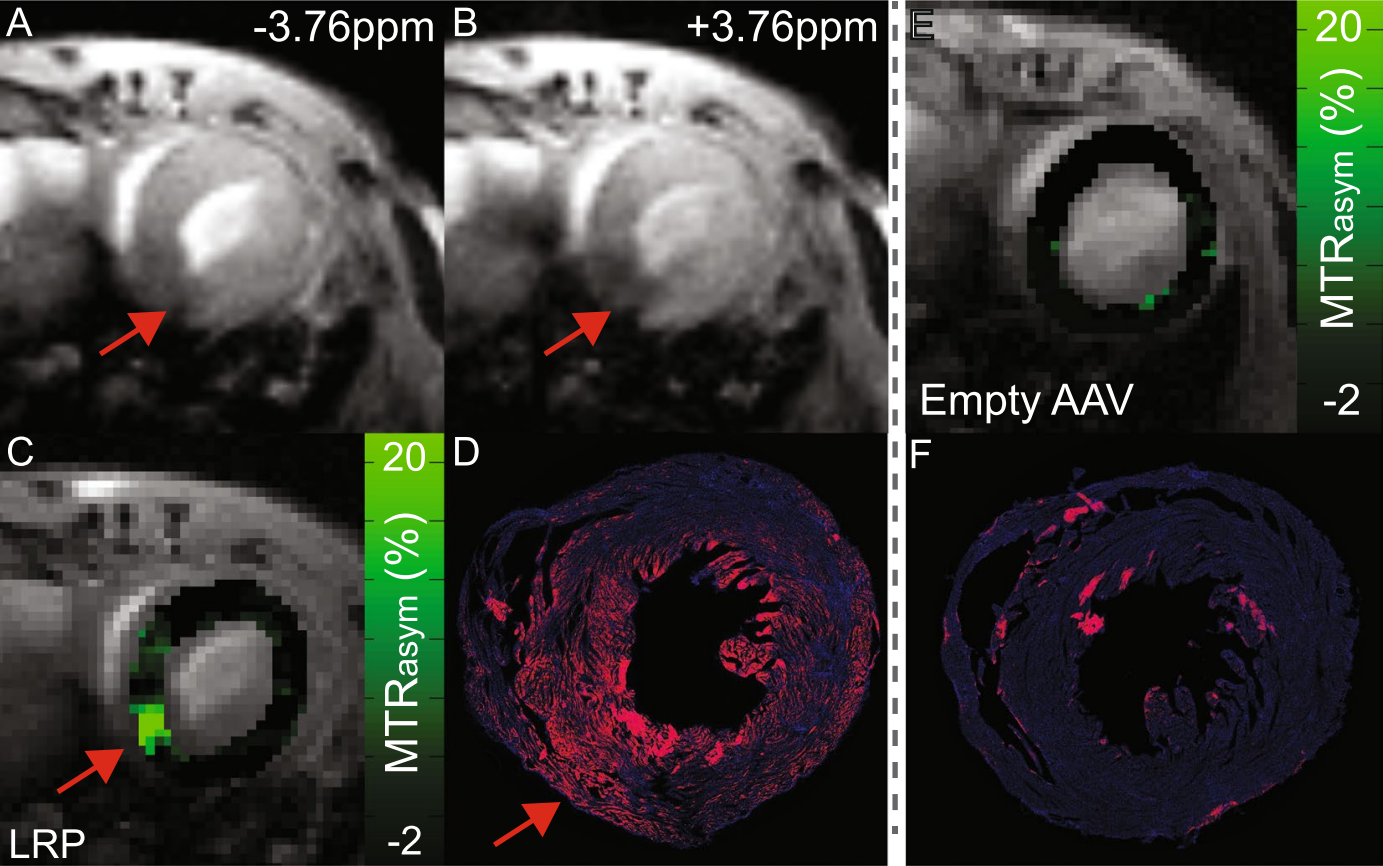
Direct injection of AAV9 encoding for the Lysine Rich Protein results in regionally specific CEST contrast at 45 days after injection. (**A**) Cardiac CEST image acquired following radiofrequency saturation at −3.76ppm (control excitation). The site of injection is indicated by the red arrow. (**B**) Corresponding cardiac CEST image acquired following saturation at +3.76ppm. The excitation of LRP and subsequent exchange of saturated magnetization results in a reduced steady state longitudinal magnetization, and thus signal intensity, in voxels containing LRP expressing cells. (**C**) CEST contrast calculated as MTR_asym_ from images A and B is elevated in the septum and inferior wall about the inferior right ventricular insertion point (red arrow). Slight elevations in MTR_asym_ are observed in the interventricular septum. (**D**) Immuno-fluorescent staining of the corresponding tissue section confirms expression of the LRP-V5 construct in the same tissue regions as observed using CEST MRI. (**E**) Injection of an empty AAV9 vector did not result in observable CEST contrast or (**F**) positive staining of isolated tissue sections. Higher magnification images of D and F are shown in Supplemental Figure 1.

In the first study arm, we sought to determine whether injection of an AAV9 vector encoding for LRP into a defined region of left ventricular myocardium would result in regionally elevated CEST contrast. Mice undergoing intra-myocardial injection of AAV9 were randomized to receive either AAV9 containing Lysine Rich Protein and V5 (n = 9) or an empty AAV9 vector (n = 6). Cardiac CEST-MRI was performed at 15 and 45 days after injection to probe for contrast generated by LRP expression. Representative cardiac CEST images and corresponding MTR_asym_ maps are shown in Fig. 2 for both vectors.

**Figure 3:**
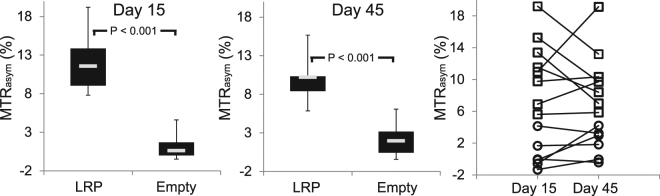
Time-course of regional changes in CEST contrast. Box and whisker plots summarize measurement of regional MTR_asym_ values at 15 and 45 days after intra-myocardial injection of either AAV9 encoding for LRP or an empty AAV9 vector (grey bar represents the mean value). At both time points, MTR_asym_ values within tissues that positively stained for V5 expression were significantly higher than corresponding regions of interest from mice receiving an empty AAV9 injection. While mean MTR_asym_ values in the AAV9-LRP group were not substantially different between days 15 and 45, (Right) individual mice within the AAV9-LRP group (squares) demonstrated diverse changes in MTR_asym_ values between both time-points. The corresponding values in mice receiving empty AAV9 vectors (circles) were consistent over time.

Immuno-fluorescent staining was used to guide the definition of regions of interest on MR images for mice receiving AAV-LRP. For mice receiving the empty AAV9 vector, hematoxylin and eosin staining was used to identify the location of the needle track in the imaged slice, and the corresponding region of myocardium was segmented on MR images. While CEST contrast measured in the myocardium of mice receiving an empty AAV9 vector was uniformly low (0.63 ± 2.0% Day 15, 1.96 ± 1.86% Day 45), the average MTR_asym_ value within V5-LRP expressing regions was significantly elevated at both 15 (11.6 ± 4.4%) and 45 (10.2 ± 3.9%) days after injection (Fig. 3). Prior to AAV9 administration the average myocardial MTR_asym_ values in the same regions of interest among all mice were 0.74 ± 2.13%. The application of the same region of interest over time among the AAV9-LRP cohort revealed temporally heterogeneous dynamics of Lysine Rich Protein CEST contrast amongst mice (Fig. 3).

### Systemic injection of AAV9-LRP heterogeneously elevates CEST contrast

**Figure 4:**
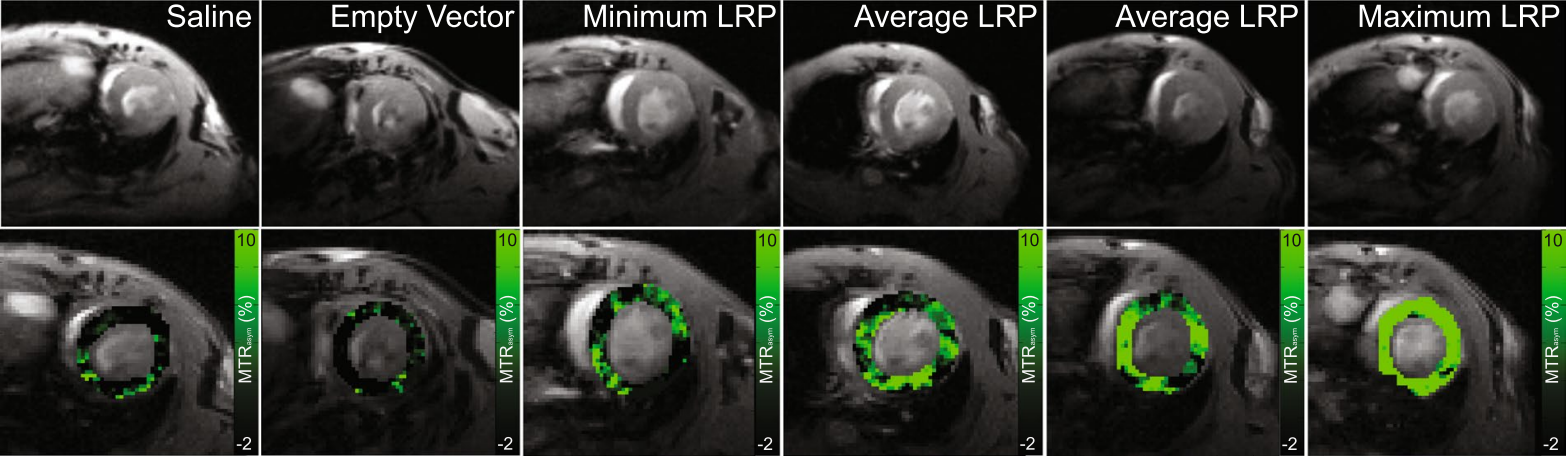
Representative maps of MTR_asym_ at 60 days after tail vein injection of either saline, empty AAV9, or AAV9-LRP. Myocardial MTR_asym_ values remained low in mice receiving either saline or an empty AAV9 vector. Among mice receiving AAV9-LRP, the average response resulted in a large number of voxels with elevated MTR_asym_ across the heart. However, variability was observed in both the spatial pattern and magnitude of MTR_asym_ elevation among mice receiving AAV9-LRP. This variability can be appreciated by comparing the MTR_asym_ maps generated in mice with the lowest change in MTR_asym_ over baseline levels (Minimum LRP), the group average changes in MTR_asym_ over baseline (Average LRP), and the maximal change in MTR_asym_ over baseline levels (Maximum LRP).

In the second arm of this study we sought to probe the time-course of cardiac CEST contrast following the systemic administration of AAV9 virus containing LRP. Mice were randomized to receive either AAV9 encoding LRP-IRES-eGFP-V5 (n = 13), an empty AAV9 vector (n = 9), or saline (n = 7), delivered intravenously through the tail vein. Cardiac CEST MRI was performed in one mid-ventricular slice at one, sixty, and ninety days after administration. Representative images and corresponding maps of MTR_asym_ from data acquired sixty days after administration are shown in Fig. 4.

**Figure 5:**
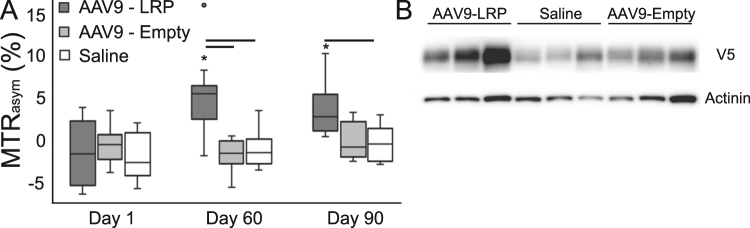
Systemic administration of AAV9-LRP. (**A**) Box and whisker plots of mean myocardial MTR_asym_ values reveal a similar absence of CEST contrast 24 hours after intravenous injection of either AAV9 or saline (−1.6 ± 3.9% AAV9-LRP, −2.1 ± 2.74% AAV9-Empty, −0.65 ± 2.3% Saline). In mice receiving AAV9-LRP the mean myocardial MTR_asym_ values increased substantially by Day 60 (4.9 ± 4.3% AAV9-LRP, −1.0 ± 2.3 AAV9-Empty, −1.87 ± 2.1% Saline) and remained elevated at 90 days after injection (3.4 ± 3.1% AAV9-LRP, −0.6 ± 2.3 AAV9-Empty, 0.1 ± 2.3% Saline) in comparison to Day 1. (**B**) Representative western blots of mouse heart samples using a V5 antibody confirmed expression of LRP-V5 in mice exposed to the AAV9-LRP construct at Day 90. The corresponding full -length gels can be found in Supplemental Figure 2. Co-IP gels demonstrating reduced non-specific binding can be found in Supplemental Figure 2. (*P < 0.05 vs. Day 1 in same group, ^_^P < 0.05 between groups at same time point).

The intravenous injection of AAV9 encoding for LRP-IRES-eGFP-V5 resulted in significant CEST contrast in the hearts of mice by sixty days after administration as compared to corresponding measurements acquired one day after administration (Fig. 5). In contrast, neither saline nor an empty AAV9 virus used as controls resulted in similar changes in CEST contrast at any point during the imaging time-course (Fig. 5). Excision and coimmunoprecipitation of hearts at ninety days after injection confirmed expression of LRP-V5 in mice receiving AAV9 encoding for LRP and V5 (Fig. 5). Comaprison of mean MTR_asym_ values to corresponding quantification of V5 expression from whole heart homogenates demonstrated a promising trend when compared to quantification by western blot analysis (Pearson’s ρ = 0.5, p = 0.14, Figure S3) and a significant correlation when compared to quantification from co-immunoprecipitation (Pearson’s ρ = 0.67, p = 0.04, Figure S3).

### Impact of LRP expression on cardiac structure, function, and hemodynamics

**Figure 6:**
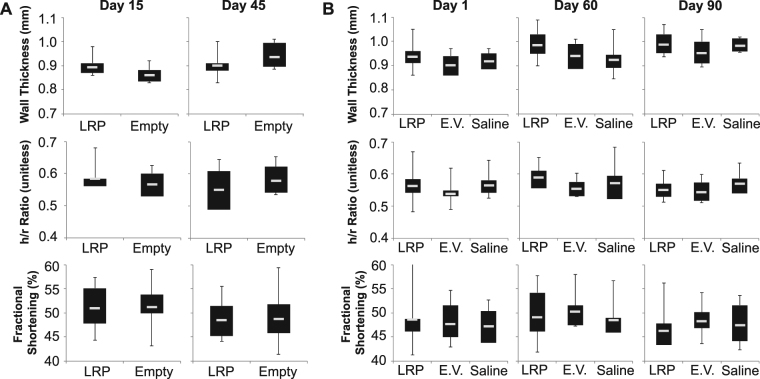
Measures of ventricular structure and function were unchanged by LRP expression status. In both the direct injection (left) and systemic administration (right) protocols ventricular structure as assessed by septal wall thickness and the ratio of wall thickness to the inner radius (h/r ratio) was not significantly different between hearts in which LRP expression was induced (LRP), empty vector (Empty, E.V.), and saline controls. Ventricular systolic function assessed as peak fractional shortening was similarly unchanged between groups and over time. Anesthesia was maintained using 1.5% isoflurane in oxygen. Mean and standard deviation values for all graphed components can be found in Supplemental Table 3.

The impact of LRP expression on cardiac structure was examined by measuring mid-ventricular end-diastolic septal thickness and the ratio of septal thickness to inner left ventricular radius (h/r ratio). Myocardial systolic function was assessed as peak fractional shortening. Neither the direct injection nor the systemic administration of AAV9-LRP resulted in changes in left ventricular structure or function at any point in the observed time-course (Fig. 6). Heart rate data for the systemic (Table S1) and direct (Table S2) administration protocols can be found in the supplemental data section. Within the systemic administration study heart rates were similar at one and sixty days after viral administration, but elevated in mice receiving saline compared to either AAV9 group at ninety days after administration. Within the direct injection protocol, heart rates were similar between AAV9 cohorts at both time points, though both groups showed significant increases in heart rate between time points.

## Discussion

Among the many challenges that remain in the field of cardiac gene therapy using viral delivery, the inability to non-invasively quantify the extent, magnitude and duration of therapeutic gene expression without radiation based imaging or biopsy approaches remains a significant obstacle. As a result, it is difficult to determine whether the eventual fruition or absence of functional outcomes is due to limited delivery, abrogated expression, or a lack of therapeutic effect despite adequate expression. In this study we demonstrated the ability to serially image longitudinal changes in CEST contrast generated by expression of a genetically encoded CEST-MRI reporter gene in the mouse heart following AAV9 mediated delivery. The results of this study have several important potential future clinical implications. First, patient examination with CEST-MRI after initiation of viral gene therapy could allow for whole heart, radiation free and non-invasive assessment of initial gene delivery and time dependent expression patterns. Second, since the contrast generated by expression of CEST active reporter genes is selectively ‘turned on’, measurement of functional outcomes of gene therapy including tissue thickness, contractile function, fibrosis, and perfusion can be assessed in series in the same imaging session. Subsequently, the coupling of gene expression patterns and therapeutic outcome can be determined on an individualized basis. This is in contrast to reporter gene methods for cardiac MRI that have used either expression of iron regulatory proteins such as Ferritin^26^ or required administration of iron oxide nanoparticles targeting genetically encoded membrane reporter proteins^27^. In both cases, assessment of gene expression requires substantial T2/T2* contrast that limits the capacity to acquire additional meaningful outcome measurements in the same tissue region. Finally, while we did not examine off-target expression of the Lysine Rich Protein, CEST-MRI is already applied to additional organs including the brain, lungs, kidneys, and skeletal muscle. Combined measurement across organ systems can allow for individualized treatment planning that targets the optimal response in the heart while minimizing off target expression.

Pre-clinical studies of AAV9 mediated gene delivery to the heart have previously used either immuno-histochemical staining of isolated tissue sections or *in vivo* bioluminescence imaging to probe for gene expression patterns^1,9,28–31^. In agreement with prior studies that utilized serial bioluminescence imaging following systemic AAV9 administration^8,9,28^, cardiac CEST contrast generated by LRP was heightened at both sixty and ninety days post administration compared to baseline levels. In a prior study by Prasad *et al*., systemic administration of AAV9 through the jugular vein of 1 week old mice led to uniform expression of eGFP throughout the ventricular myocardium within 30 days^9^. In contrast, we observed spatially heterogeneous CEST contrast across the myocardium in mice receiving systemic injection of AAV9-LRP (Fig. 4). Similar spatial heterogeneity was also observed at 70 days after systemic injection of AAV9 by Werfel *et al*. upon staining of isolated tissue sections for the LacZ reporter^28^. Differences in observed patterns between studies may be attributed to the age of mice at time of exposure (1 week vs. 8 weeks Werfel *et al*. and current study), the promoters used to drive expression of the reporter gene (cTnT vs. CMV in current study), and route of administration (jugular vs. tail vein Werfel *et al*. and current study). Interestingly, the gradients in CEST contrast observed in most mice receiving AAV9-LRP seemed to follow a coronary distribution pattern (Fig. 5, Average LRP), suggesting heterogeneous delivery following intravenous administration. Similar concerns regarding non-uniform delivery following intravenous administration have been raised by others^10,28,29^. However, additional studies that directly compare regional CEST contrast to corresponding quantitative measures of tissue LRP levels are necessary to clarify whether these patterns are truly reflective of underlying LRP expression or represent a threshold level of LRP expression that is required for CEST detection. Regrettably, we utilized all of the heart tissue from this study arm after Day 90 to perform a co-immunoprecipitation assay in order to confirm expression of the V5 tag and are unable to examine the regional correlation between CEST contrast and LRP expression. However, correlation analysis between MTR_asym_ at Day 90 and the V5 western and IP signals measured from whole heart homogenates demonstrated strong correlation between CEST-MRI contrast and underlying LRP expression (see Supplemental Figure 2). A direct correlation between CEST contrast and regionally constrained V5 western signals as part of future studies will more directly enable examination of the correlation between the molecular and the imaging system components. In addition, while we have previously shown that B0 inhomogeneity across the mouse heart is smaller than the bandwidth of the saturation pulse used to generate CEST contrast^24,32^, it is important to note that B1 inhomogeneity of the saturation pulse can lead to regional differences in observed CEST contrast that may not obscure underlying changes in LRP expression.

In comparison to the heterogeneous patterns observed following systemic administration, direct injection of AAV9-LRP into the myocardium produced robust CEST contrast about the site of injection (Fig. 2). This finding agrees with prior large animal models that used the Na/I transporter to examine the impact of delivery route on subsequent gene expression. Catheter based delivery into the myocardium of pigs by Moulay *et al*. produced robust contrast on SPECT/CT images, while contrast in the heart following intravenous administration was considerably attenuated^10^. In a prior study by Prasad *et al*., serial *in vivo* bioluminescence imaging following intra-myocardial injection of AAV9 in adult mice revealed a rapid increase in luciferase expression by 14 days after administration that thereafter remained comparatively constant until 42 days after injection^8^. In the current study the average CEST contrast measured in the same regions of interest over time in mice receiving a direct injection of AAV9-LRP was not significantly different as a group between 15 and 45 days post injection (Fig. 3). However, CEST contrast decreased in four mice and increased in two mice between time points. Differences in promoters (cTnT vs. CMV) and surgical techniques (open chest vs. pop out) may have contributed to the observed differences between our study and that of Prasad *et al*.^8^. Importantly, *in vivo* bioluminescence measures of average radiance represent a complex function of integrated luciferase expression over the entire heart combined with position dependent light scattering and attenuation. As such, changes in the spatial patterns and magnitudes of luciferase expression over time may be masked by measurement of the average radiance over the entire heart. In contrast, repeated measures of MTR_asym_ in defined and maintained regions of interest provides anatomically specific quantitation of changes in CEST contrast generated by reporter gene expression. The capacity to repeatedly make such measurements will be useful to applications that seek therapeutic gene expression in distinct regions of myocardium, e.g. restricted expression in border zone cardiomyocytes after myocardial infarction, but previously relied upon *ex vivo* tissue staining to confirm spatial localization.

The design of the current iteration of LRP raises several potential limitations to both the ultimate sensitivity of this method and to its clinical translation. For example, the repetitive nature of the LRP can lead to instability both at the transcriptional and translational level. Repetitions in the DNA can lead to recombination and exclusion of fragment of the genes. This issue can be addressed by re-engineering and optimizing the DNA sequence as was done for the human protamine-1^33^. At the protein level, replacement of some of the lysine residues in strategic location can minimize the charge and reduce cleavage by intracellular proteases. It is important to note that enzymatic cleavage of LRP into shorter peptides will not impact the total measured CEST contrast, because the contrast is not determined by the structure of the LRP but rather by the total number of exchangeable protons. Newer versions of LRP that are under development and utilize a more sophisticated design of the amino acid sequence to reduce the likelihood of enzymatic cleavage will boost the contrast generated by constitutive expression of LRP. In parallel, it is unknown whether expression of substantial quantities of LRP will alter the intracellular environment due to the strong positive charge of Lysine, and whether such alterations will negatively impact cellular function. Early studies performed by Gilad and colleagues in which LRP was expressed in cancer cells did not demonstrate any changes in cellular proliferation or viability^18,21^. Although extrapolation of this finding to non-proliferative cells including cardiomyocytes is not straight forward, we did not observe any detrimental effect of LRP expression on cardiac structure or function in mice. Importantly, we did not perform any experiments on isolated cardiac tissue to probe for markers of cellular remodeling or stress. The inclusion of experiments in which LRP is expressed in induced cardiomyocytes derived from human induced pluripotent stem cells would be required in future studies to more thoroughly evaluate the safety profile of LRP. In addition, we were unable to probe diastolic function or changes in systolic function with stress that manifest earlier than changes in resting systolic function. Future studies will examine such metrics to more thoroughly examine the impact of LRP expression of cardiac function. Finally, the capacity to differentially quantify a small fraction of cells expressing a therapeutic gene at high levels as opposed to a large fraction of cells expressing a therapeutic gene at low levels will be crucial for optimization of viral vector design and delivery. Since CEST contrast from LRP is generated by the exchange of saturated protons from LRP to the surrounding cytosol water, and the MTR_asym_ values in each voxel represent the aggregate impact of all cells within the voxel, the fraction of successfully transduced cells likely dominates the overall CEST contrast that is observed. This is supported by comparison of the CEST-contrast measured by Gilad *et al*.^18^, where a bolus of LRP-expressing cells extended over several voxels, to that of Farrar *et al*., where only a sub-fraction of cells within a voxel expressed LRP^22^. Similarly, beyond using immuno-fluorescence to guide the definition of regions of interest for analysis, we did not perform any additional quantification of transfection efficiency. The presence of non-specific elevation in both MTR_asym_ and immuno-fluorescence images observed in negative control animals indicates that measurement of low level early transfection or persistence of low intensity signal over time must be further validated beyond CEST-MRI measurements. However, it is crucial that future studies examine the balance that exists between both components and whether this CEST-MRI approach can differentiate the level of LRP expression within cells from the overall fraction of cells expressing LRP in a given voxel.

The ability to non-invasively and repeatedly image gene expression patterns and functional outcomes in parallel will serve an important role in the continued development of cardiac gene therapy. Current efforts at optimization of delivery mechanisms^29,30^, novel formulae for viral preparation by inclusion of different concentrations of empty capsids^7^, or targeting with tissue specific promoters^34^ can be aided by the inclusion of genetically encoded CEST-MRI reporter genes such as LRP. With the eventual development of either FDA approved versions of LRP, or through the use of a coupled membrane-bound reporter peptides and targeted CEST molecules^35^, the physiologically rich data sets that can be acquired could lead to individualized planning, treatment, and follow up to maximize outcomes.

## Materials and Methods

### Generation of viral vectors

The plasmid pIRES-EGFP-LRP204 was a gift from Assaf Gilad. The plasmids pZac2.1 and pAdDF6 are gifts from Paul Murphy (University of Kentucky). The LRP gene was designed *de vovo* form eight 83-base-pair-long synthetic oligonucleotides^18^ and cloned directly into the plasmid pIRES-EGFP (Clontech). The plasmid pAAV9 was obtained from the University of Pennsylvania Viral Core. The plasmid pSMPUW-IRES-EGFP and 293LTV cells were purchased from Cell Biolabs. The vector for expressing V5-tagged LRP204 in recombinant AAV9-serotyped adeno-associated virus was constructed by ligating the NheI/NotI fragment of pIRES-EGFP-LRP204 into similarly-digested pZac2.1 to create MVAp150213B. DNA sequencing indicated that this construct only retained a subset (50) of the lysine repeats. The full complement of repeats was restored by ligating a 690 bp NheI/BamHI fragment of pIRES-eGFP-LRP204 into similarly digested MVAplan150213B to create MVAp150302D1 [pAAV CMV_prom_::LRP204-IRES-GFP]. The V5 epitope was then added to the amino-terminus of LRP204 by ligating an adaptor constructed by hybridizing the oligonucleotides CTAGCCACCATGGGTAAGCCTATCCCTAACCCTCTCCTCGGTCTCGATTCTACTCGAG and GATCCTCGAGTAGAATCGAGACCGAGGAGAGGGTTAGGGATAGGCTTACCCATGGTGG into BglII/NheI digested MVAp150302D1 to create MVAp150506A5 [pAAV CMV > V5:200K_IRES_EGFP]. The vector for constructing the empty, control AAV was constructed by ligating the 1349 bp EcoRI/SalI fragment carrying the IRES-GFP from pSMPUW-IRES-EGFP into EcoRI/SalI-digested pZac2.1 to create ViCo1.28 [pAAV CMV_prom_::LRP204-IRES-GFP]. The fidelity of each construct was confirmed by DNA sequence analysis. AAV9-pseudotyped viruses were prepared by co-transfecting 10 T225 culture flasks of 293LTV cells with 250 mg pAAV2/9, 500 mg pAdDF6 and either 250 mg of MVAp150506A5 or the empty vector, ViCo1.28 using 5 mg polyethyleneimine to enhance DNA uptake. After three days, the cells were harvested, washed, suspended in 13 ml 150 mM NaCl, 50 mM Tris·Cl pH 8.4, 0.5% deoxycholate and 50 U/ml of benzonase and incubated at 37 °C for 30 min. An additional 2.8 ml 5 M NaCl was added and the incubation was continued for another 30 min. at 45 °C. The cell suspension was then subjected to four freeze/thaw cycles (30 min at −80 °C/30 min at 45 °C). The lysate was then partially clarified by centrifugation at 18,500 × g for 10 min at 20 °C. The supernatant was laid on top of an iodixanol step gradient and centrifuged at 350,000 × g for 1 hour at 18 °C. The interface between the 40% and 54% iodixanol layers was withdrawn and spin-purified and concentrated using four washes with PBS in an Amicon Ultra-15 100,000 MWCO spin concentrator. The virus preparation was then titered using quantitative PCR with primers directed against the CMV promoter region of the DNA encapsulated in the virions.

### Administration of AAV9

A total of 45 male, 8 week old, C57B6/J mice were purchased from Jackson Labs. Mice were randomly assigned to either a systemic injection protocol, or a direct injection protocol. All animal studies adhered to the NIH guide for the care and use of laboratory animals and were approved by the Animal Care and Use Committee at the University of Kentucky. For systemic administration, approximately 100 μL of AAV9 containing LRP-V5 (n = 13), an empty AAV9 vector (n = 10), or saline (n = 7) were injected via the tail vein. The viral titter for both AAV9 vectors was 1.93 × 10^13^ genomes/mL corresponding to a dose of approximately 1.93 × 10^12^ genomes per injection. For the direct injection protocol 25–30 μL of either AAV9 containing LRP-V5 (n = 9) or an empty AAV9 vector (n = 6) were injected directly into the free wall of the left ventricle. The viral titter used in this arm was 2.8 × 10^13^ genomes/mL resulting in approximately 0.7 × 10^12^ viral genomes per injection. Intra-myocardial injection was accomplished using the pop-out technique as described by Gao *et al*.^36^. Briefly, mice were anesthetized using 5% isoflurane, an incision was made in the skin and the pectoralis muscles were separated. Afterwards, a puncture was made in the third intercostal space and the heart was gently pushed out of the body. A 29 g needle was inserted at the apex and advanced towards the mid-ventricle, after which the viral vector was injected directly into the myocardial mid-wall. The site of injection was visually confirmed by bleaching of the myocardium tissue at the time of injection. Afterwards, the heart was returned to the thoracic cavity, 8–0 suture was used to close the intercostal space, and mice were allowed to recover in room air.

### Magnetic Resonance Imaging

Imaging was performed on a 7 T horizontal scanner (ClinScan, Bruker Biospin MRI GmbH, Ettlingen, Germany) using a cylindrical volume coil for excitation (outer diameter = 11.2 cm, inner diameter = 8.6 cm) and 4-channel phased array surface coil for detection. Anesthesia used 1.5% isoflurane in oxygen and body temperature was maintained (37 °C) using circulating water. Electrocardiogram (ECG) and respiratory gating used a Small Animal Instruments (SAI, Stonybrook, NY) system. The MRI pulse sequence used to generate CEST images has been described in detail in Pumphrey *et al*.^24,25^. The acquisition of cine images was electrocardiogram and respiratory gated, with dummy excitation pulses maintaining steady state conditions. Imaging parameters included FOV = 2.5 × 2.5 cm, Matrix = 256 × 128, true in-plane resolution = 200 × 100 μm, slice thickness = 1 mm, TR/TE = 6.92/3.42 ms, 4 averages, and 15° flip angle. For CEST-saturation a train of spatially non-selective Gaussian pulses (8.8 ms, 200 Hz bandwidth, 1 ms inter-pulse delay, number of pulses = 196, flip angle = 360°, B1average = 4 μT) were applied at offset frequencies of ± 3.76 ppm. The total acquisition time was 2.5–3 minutes per cine image, or 5–6 minutes per pair. For mice included in the systemic administration protocol, one mid-ventricular slice was consistently imaged at one, sixty, and ninety days post AAV9/saline administration. In the direct injection protocol one slice slightly apical to the mid-ventricle was imaged prior to AAV9 injection. At fifteen and forty five days following AAV9 injection, three slices were imaged across a 3 mm slab encompassing the mid-ventricle, a slightly apical slice, and a slightly basal slice. For all protocols the distance for all imaged slices from the left ventricular apex and mitral valve plane were recorded at the time point of initial imaging and used to replicate the same slice positions over time. At the conclusion of each study mice were anesthetized in 5% isoflurane and perfused with saline solution for two minutes. Hearts were excised and flash frozen in liquid nitrogen.

### Co-immunoprecipitation (Co-IP), and Western Blotting (WB)

Hearts were homogenized using homogenization buffer containing 1 × Hsaio TBS, PMSF (1:1000), 1:1000 Protease Inhibitor cocktail set 3 (EMD Millipore #539134), 1:1000 PhosphotaseArrest II (GBioscience #786-451), and 1:1000 PhosphotaseArrest III (GBioscience #786-452). Samples were centrifuged at 10,000 rpm and supernatant was isolated. Protein supernatant concentration was determined using BCA (Thermo #23225). Samples were incubated with anti-V5 antibody (Abcam #ab184144) at 1:1000 overnight at 4 °C. G-protein beads were then incubated with samples for 4 hours. Samples were centrifuged at 3000 rpm and beads were isolated. Beads were washed twice and centrifuged at 3000 rpm. Beads were centrifuged at 10,000 rpm and supernatant was entirely removed. Beads were then resuspended in 2× Laemmli Buffer with beta-mercaptoethanol. Samples were run on 10% Tris-Glycine gels (BioRad #5671034), and transferred to PVDF membranes (EMD Millipore #IPVH00010). Primary antibodies used were Anti-V5 antibody (Abcam #ab184144) at 1:1000 and Actinin (Sigma-Aldrich #A2066) at 1:1000. Protein signals were revealed by ECL (Pierce #32106) using a GE Amersham Imager 600UV system. Image analysis was performed using ImageJ. Bands of interest were normalized to a loading control. Statistical analysis of the bands was performed using Student’s t-test in GraphPad Prism.

### Immunofluorescence

Immunofluorescent staining was performed on 6–10 axial heart slices spanning the left ventricle using a previously described protocol^37^. Briefly, tissue was mounted in 30% ethanol on ColorFrost Plus slides (Thermo, cat# 9951APLUS-006) and then blocked in blocking buffer (10% normal goat serum, 0.2% Triton X-100, 0.02% 10% sodium azide in 1X tris buffered saline). Primary antibodies were suspended in blocking buffer and incubated at 4 degrees Celsius overnight. Slides were then washed and incubated in secondary antibodies (Alexafluor Goat anti-Rabbit 594 #A11037, Alexafluor Goat anti-Mouse 488 #A11029). Slides were coverslipped and then imaged. Primary antibodies used were Anti-V5 antibody (Abcam #ab184144) at 1:100.

### Microscopy

A BioTek Cytation5 Imaging Multi Mode Reader was used to capture images of stained hearts. All immunolabeling acquisition intensities and microscope settings were kept consistent across all images. Montage scans were taken of entire heart sections within similar regions. Images were prepared using Photoshop Cs6 (Adobe) and Illustrator Cs6 (Adobe).

### MRI Data Analysis

Data analysis was performed in MATLAB R2012b (Mathworks, Nattick, MA) using custom built software. For each mouse and time point the end-diastolic image acquired following saturation at 3.76 ppm was registered to the corresponding image acquired following saturation at −3.76 ppm. Maps of the asymmetric magnetization transfer ratio (MTR_asym_) were then calculated on a voxel-wise basis as MTR_asym_ = (S_−3.76ppm_ − S_3.76ppm_)/S_−3.76ppm_ * 100. Endocardial and epicardial borders of the myocardium were defined using images acquired following saturation at −3.76 ppm. The average myocardial MTR_asym_ value was measured for the systemic administration protocol. For analysis of data generated from mice in the direct injection model receiving AAV9-LRP-V5, the mean MTR_asym_ value across a region of interest defined as V5-positive based on the matching mid-ventricular immunofluorescence staining pattern was measured. For mice receiving empty AAV9, the mean MTR_asym_ value along the area of the LV free wall about the needle track from H&E stained images was measured. Across all mice, fractional shortening was measured at the mid-ventricle as FS = (diastolic diameter − systolic diameter)/diastolic diameter. The ratio of septal wall thickness to chamber radius was also measured at the mid ventricle. For all figures shown, maps of MTR_asym_ are processed such that only voxels from the left ventricle are superimposed upon corresponding magnitude reconstructed images.

### Statistical Analysis

Statistical analysis was performed in SigmaPlot v13 (Systat, San Jose, CA). Two way analysis of variance (ANOVA) tests were used to assess differences in MTR_asym_, cardiac structure and function, and heart rate between groups of mice at each time point and within groups of mice across time points. Holm-Sidak was used for pairwise comparison. Unless otherwise noted all data was normally distributed. Statistical significance was defined as p < 0.05. Data is graphically represented throughout the manuscript using box and whisker plots. In places where data is numerically represented, the mean ± standard deviation is presented.

### Data and materials availability

All data is maintained on a server at UC Berkeley and will be made available to interested researchers upon request. Interested parties should contact corresponding author.

## Electronic supplementary material

Supplemental Data and Images
