# Supplementary material for: Non-invasive detection of adeno-associated viral gene transfer using a genetically encoded CEST-MRI reporter gene in the murine heart
Source: Sci Rep. 2018 Mar 15;8:4638. doi: 10.1038/s41598-018-22993-4 (PMC5854573; doi:10.1038/s41598-018-22993-4)
Supplement: Supplementary file 1 — Supplemental Data and Images [file 41598_2018_22993_MOESM1_ESM.docx]

­­Non-invasive detection of adeno-associated viral gene transfer using a genetically encoded CEST-MRI reporter gene in the murine heart

**Authors:** Shelby Meier^1,2^, Assaf A Gilad^3,4,5,6,7^, J. Anthony Brandon^1^, Chenghao Qian^1^, Erhe Gao^8^, Jose F Abisambra^1,2^, Moriel Vandsburger^9*†^

**Affiliations:**

^1^ Department of Physiology, University of Kentucky, KY, USA.

^2^ Sanders Brown Center on Aging, University of Kentucky, KY, USA.

^3^ Department of Radiology, Johns Hopkins University, MD, USA.

^4^ The Institute for Cell Engineering, Johns Hopkins University, MD, USA.

^5^Department of Biomedical Engineering, Michigan State University, MI, USA

^6^The Institute of Quantitative Health Science and Engineering, Michigan State University, MI, USA

^7^Department of Radiology, Michigan State University, MI, USA

^8^ Center for Translational Medicine, Temple University, PA, USA.

^9^ Department of Bioengineering, University of California, Berkeley, CA, USA.

*All correspondence should be addressed to Moriel Vandsburger at moriel@berkeley.edu

†Corresponding address: 281 Hearst Memorial Mining Building, Berkeley, CA, 94707

Supplemental Data


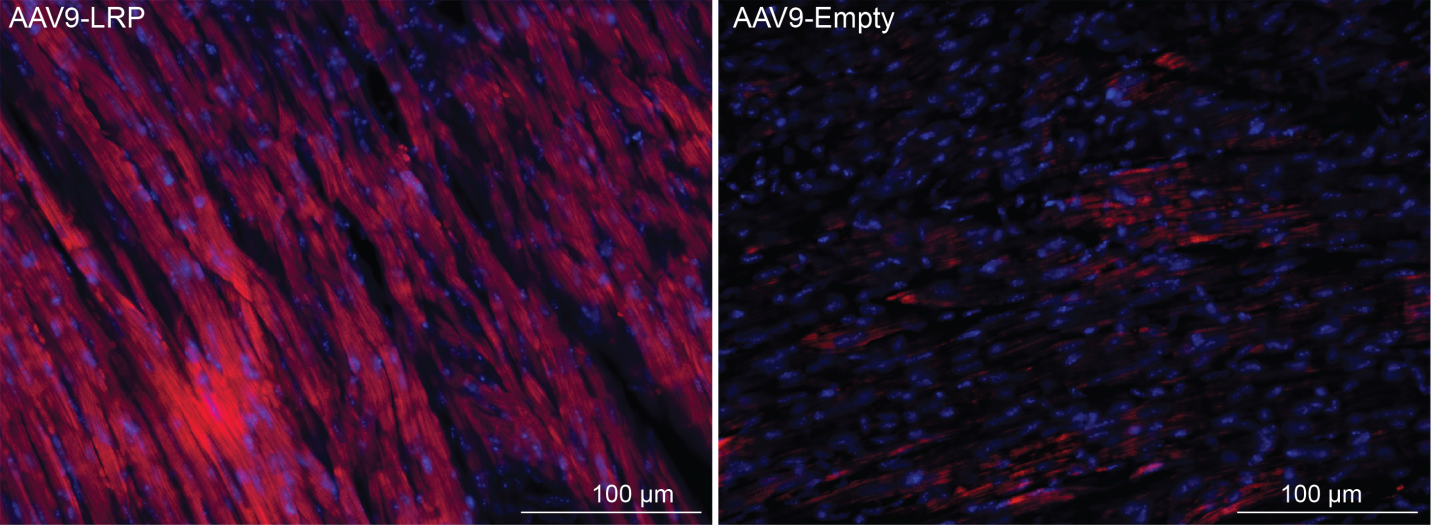


**Figure S1.** 20x magnification of the inferoseptal region in Figure 2D demonstrates positive V5 staining along myofibrils in the AAV9-LRP heart. A corresponding image, also of the inferoseptal region, from the AAV9-Empty heart shown in Figure 2F shows limited background staining for V5.


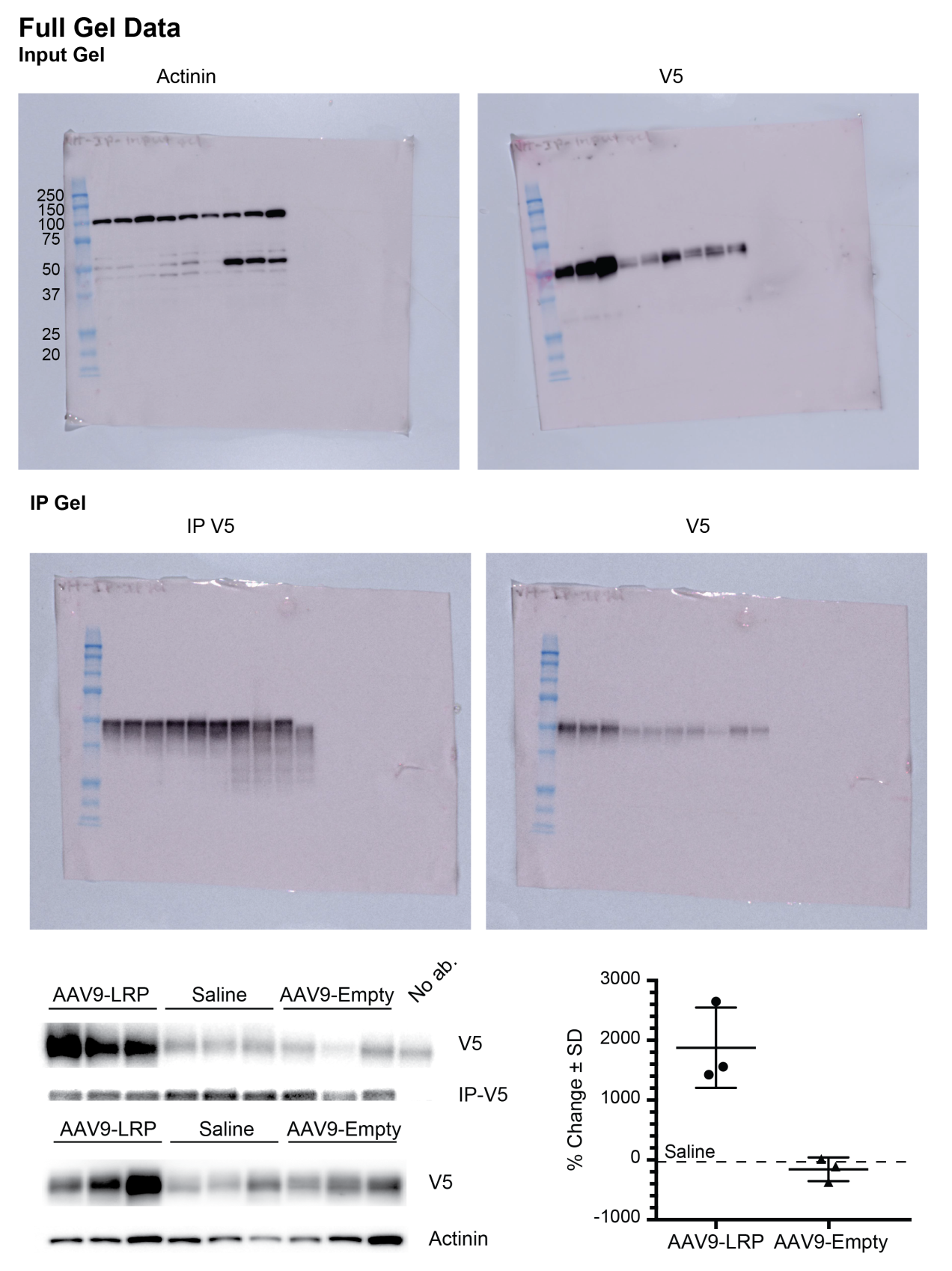


**Figure S2.** Full gel images for cropped gel data shown in Figure 5b. All gels used the same ladder. Additional Co-IP blots are shown demonstrating the reduction in non-specific staining for V5.


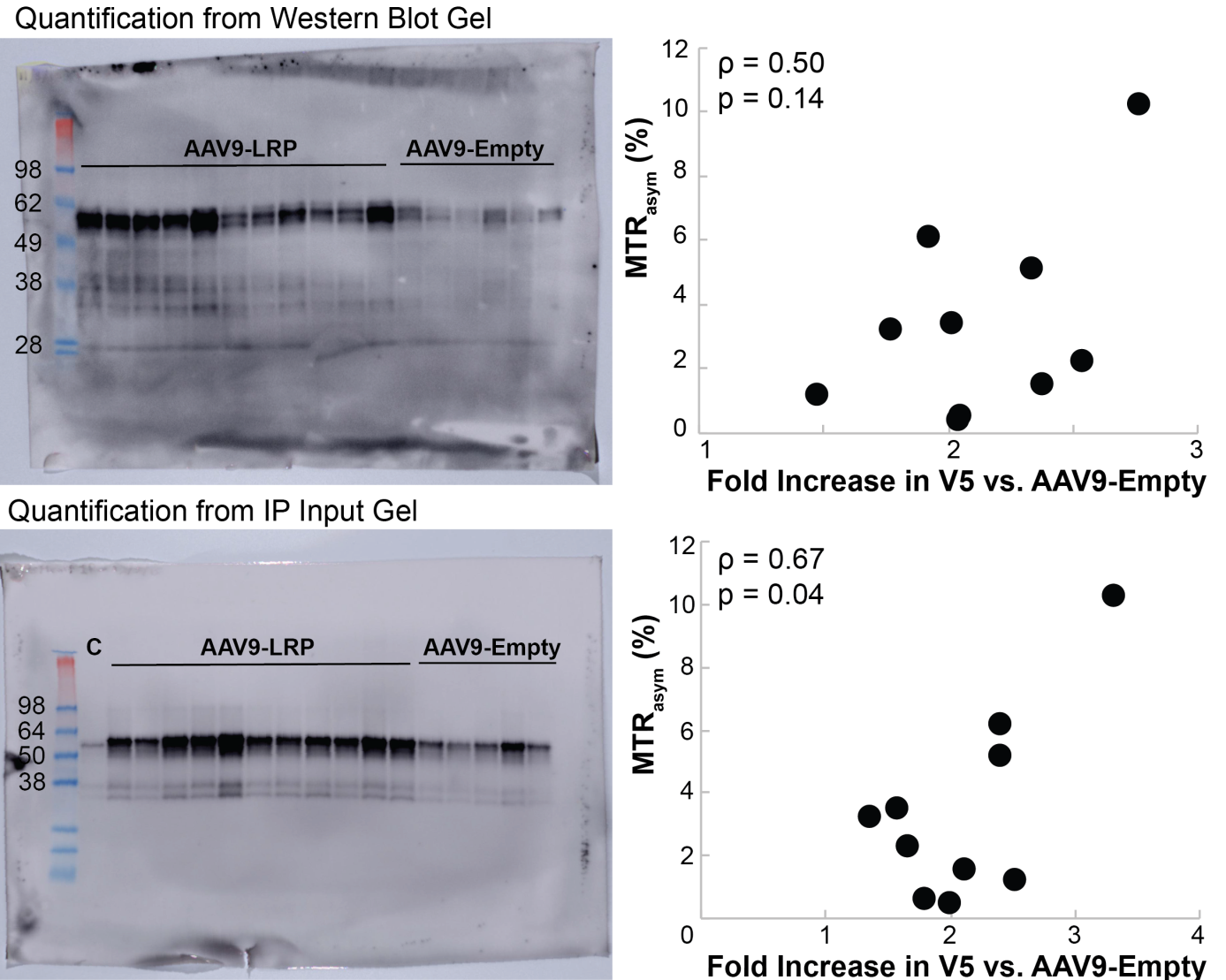


**Figure S3. Correlation of regional MTR_asym_ to whole heart V5 signal determined by immunohistochemistry. (Top)** Western blot from whole heart homogenates at 90 days after intravenous administration of either AAV9-LRP or AAV9-Empty shows strong V5 staining (~55 Kd) in mice receiving AAV9-LRP. Each V5 band was first normalized to the corresponding band in a Ponceau gel in order to normalize for total protein content. The average value of this ratio was calculated across all AAV9-Empty samples, and each normalized AAV9-LRP value was then divided by the calculated average AAV9-Empty ratio in order to calculate the fold increase in V5 vs. AAV9-Empty. Pearson’s correlation analysis revealed a moderate correlation between MTR_asym_ values at the mid-ventricle and magnitude of LRP expression across the entire heart. **(Bottom)** Similar analysis was performed on IP data in order to reduce the potential impact of non-specific binding observed in the Western blot. Changes in LRP levels were quantified by normalizing the V5 band intensity for each AAV9-LRP sample to the average intensity of corresponding bands in AAV9-Empty hearts. The lane labeled ‘C’ contains homogenates of transfected cells. Pearson’s correlation revealed a strong and significant correlation between the increase in LRP levels in whole heart homogenates and corresponding increases in mid-ventricular MTR_asym_ values. It is important to note that MTR­_asym_ values were only obtained in one mid-ventricular slice, whereas IHC quantification was performed on whole heart homogenates that include the imaged slice.

|  | Day 1 | Day 60 | Day 90 | Within Group |
| --- | --- | --- | --- | --- |
| AAV9-LRP | **535 ± 46**  P = 0.12 vs. AAV9-Empty | **520 ± 30**  P = 0.14 vs. AAV9-empty | **539 ± 31**  P = 0.054 vs. AAV9-Empty | P = 0.33 Day 1 vs. Day 60  P = 0.15 Day 60 vs. Day 90 |
| AAV9-Empty | **508 ± 23**  P = 0.25 vs. Saline | **540 ± 28**  P = 0.99 vs. Saline | **508 ± 34**  P < 0.01 vs. Saline | P = 0.02 Day 1 vs. Day 60  P = 0.054 Day 60 vs. Day 90 |
| Saline | **534 ± 58**  P = 0.94 vs. AAV9-LRP | **541 ± 36**  P = 0.19 vs. AAV9-LRP | **586 ± 21**  P < 0.01 vs. AAV9-LRP | P = 0.06 Day 1 vs. Day 90  P = 0.02 Day 60 vs. Day 90 |

**Table S1. Heart rate data for the systemic administration study.** Heart rates were similar amongst groups at one and sixty days after intravenous administration of either AAV9-LRP, AAV9-Empty, or saline. At ninety days after administration heart rate was significantly higher in mice receiving saline as compared to either group that received AAV9. However, the average heart rate among mice receiving saline was also significantly higher at ninety days after injection as compared to either prior time point. Similarly, mice receiving AAV9-Empty demonstrated higher heart rate at sixty days after injection as compared to either surrounding time point. Statistical analysis was performed using 2-way repeated measures ANOVA to compare differences between groups within each time point and differences within groups across different time points.

|  | Day 15 | Day 45 | Within Group |
| --- | --- | --- | --- |
| AAV9-LRP | **533 ± 36** | **566 ± 22** | P = 0.03 |
| AAV9-Empty | **510 ± 28** | **549 ± 20** | P = 0.02 |
| Within Day | P = 0.22 | P = 0.13 |  |

**Table S2. Heart rate data for direct injection study.** Heart rates were similar between groups at each imaging time point following direct injection of either AAV9-LRP or AAV9-Empty. Both groups demonstrated higher heart rates at forty five days after injection of AAV9 compared to fifteen days after injection.

|  | Direct Injection Protocol | | | | Systemic Injection Protocol | | | | | | | | |
| --- | --- | --- | --- | --- | --- | --- | --- | --- | --- | --- | --- | --- | --- |
|  | Day 15 | | Day 45 | | Day 1 | | | Day 60 | | | Day 90 | | |
|  | AAV9-LRP | AAV9-Empty | AAV9 -LRP | AAV9-Empty | LRP | EV | S | LRP | EV | S | LRP | EV | S |
| Wall Thickness (mm) | 0.89 ± 0.04 | 0.86 ± 0.04 | 0.90± 0.05 | 0.94 ± 0.07 | 0.94 ± 0.06 | 0.90 ± 0.06 | 0.92 ± 0.05 | 0.99 ± 0.07 | 0.94 ± 0.05 | 0.92 ± 0.08 | 0.99± 0.06 | 0.96± 0.06 | 0.98± 0.03 |
| h/r | 0.58 ± 0.04 | 0.57 ± 0.05 | 0.55± 0.07 | 0.58 ± 0.06 | 0.60 ± 0.05 | 0.54 ± 0.04 | 0.57 ± 0.05 | 0.58 ± 0.03 | 0.55 ± 0.03 | 0.57 ± 0.06 | 0.55 ± 0.03 | 0.54± 0.04 | 0.58± 0.05 |
| F.S. (%) | 51 ± 5 | 51 ± 6 | 49 ± 5 | 49 ± 7 | 49 ± 5 | 48 ± 5 | 47 ± 4 | 49 ± 5 | 50 ± 5 | 49 ± 4 | 46 ± 5 | 48 ± 4 | 47 ± 7 |

**Table S3. Mean and standard deviation values for all graphs shown in Figure 6.**
